# The extent of environmental and body contamination through aerosols by hydro-surgical debridement in the lumbar spine

**DOI:** 10.1007/s00402-017-2668-0

**Published:** 2017-03-20

**Authors:** David Putzer, Ricarda Lechner, Debora Coraca-Huber, Astrid Mayr, Michael Nogler, Martin Thaler

**Affiliations:** 1 Department of Orthopaedic Surgery, Experimental Orthopaedics, Medical University Innsbruck, Innrain 36, 6020 Innsbruck, Austria; 2 Department of Orthopaedic Surgery, Medical University Innsbruck, Anichstrasse 35, 6020 Innsbruck, Austria; 3 Division of Hygiene and Medical Microbiology, Department of Hygiene, Microbiology, and Social Medicine, Medical University Innsbruck, Fritz-Pregl-Straße 3, 6020 Innsbruck, Austria

**Keywords:** Infection, Spine, Surgical debridement, Hydro-surgery device, Contamination operating room

## Abstract

**Introduction:**

Surgical site infections occur in 1–6% of spinal surgeries. Effective treatment includes early diagnosis, parenteral antibiotics and early surgical debridement of the wound surface.

**Materials and methods:**

On a human cadaver, we executed a complete hydro-surgery debridement including a full surgical setup such as draping. The irrigation fluid was artificially contaminated with *Staphylococcus aureus* (ATCC 6538). Surveillance cultures were used to detect environmental and body contamination of the surgical team.

**Results:**

For both test setups, environmental contamination was observed in an area of 6 × 8 m. Both test setups caused contamination of all personnel present during the procedure and of the whole operating theatre. However, the concentration of contamination for the surgical staff and the environment was lower when an additional disposable draping device was used.

**Conclusions:**

The study showed that during hydro-surgery debridement, contaminated aerosols spread over the whole surgical room and contaminate the theatre and all personnel.

## Introduction

Surgical debridement is the gold standard in the case of surgical site infection of the spine. There are numerous surgical wound debridement techniques, such as debridement with scalpel, ultrasound disruption of debris and high-pressure hydro-surgery of the wound surface [10]. Debridement procedures may assist healing by removing necrotic or infected tissue, and therefore reducing the bacterial load on the wound surface [2, 3]. Non-removed infected tissue may serve as a source of antibiotic resistance and re-infection, and thereby prolong wound healing, possibly causing complications associated with the infection [1, 4, 17, 28].

High-pressure pulsed lavage systems allow a quicker and more efficient delivery of irrigant to the wound and have a superior dilutional effect which promote greater bacterial clearance [5]. Tabor et al. showed that bacterial levels were reduced by a mean of 44% (SD 50%) by bulb irrigation and 70% (SD 10%) by high-pressure pulsed lavage [24]. In a study by Hassinger et al., it has been shown that high-pressure pulsed lavage causes deeper penetration of bacteria in the soft tissue than low-pressure lavage [13].

It has already been shown that during surgical procedures, aerosols can spread over the operating room and contaminate the environment and all persons [19–21]. These aerosols, which are possibly contaminated with bacterial, fungal or viral agents, can cause infection when they come into contact with mucous membranes or small wounds or when they are inhaled by healthcare workers and the patient during the procedure [8, 9]. At our department, high-pressure hydro-surgery (Versajet, Smith & Nephew, Memphis, TN, USA) is commonly performed in cases of infection following procedures using a posterior approach to the spine. The first aim of this study was to evaluate the degree of contamination of the operating room and the surgical staff after high-pressure hydro-surgery debridement of the wound surface. The second aim was to investigate whether contamination of the operating team and room can be reduced by using additional draping devices.

## Methods

**Fig. 1:**
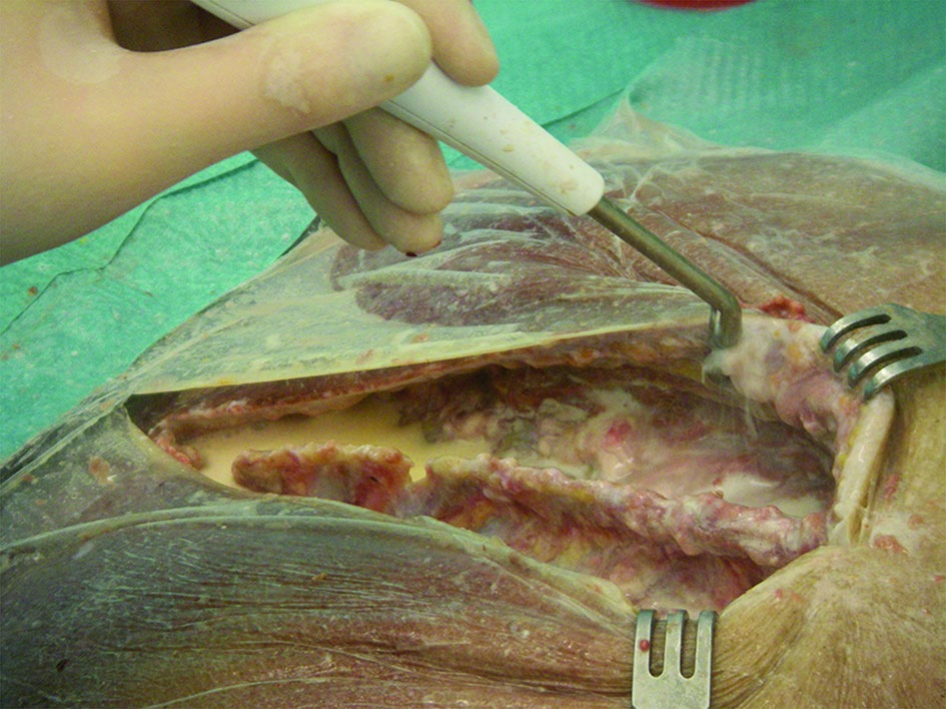
Photo showing debridement with the hydro-surgery device

**Fig. 2:**
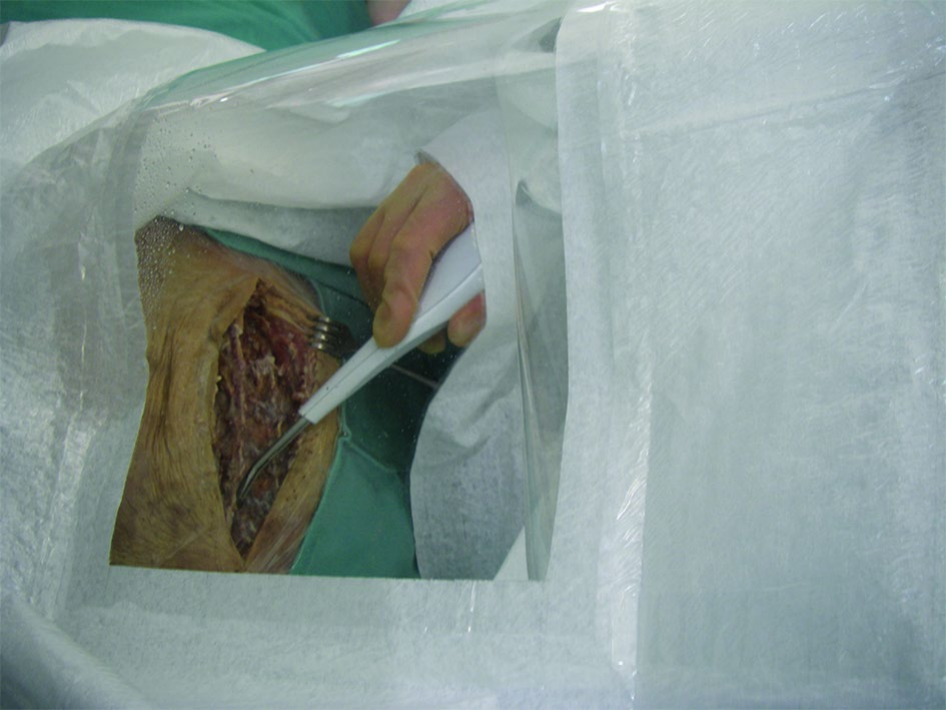
Photo showing debridement with the hydro-surgery device under the disposable draping device (tent)

In the current study, we used a standardised, already published test setup to determine aerosol contamination of operating team and room [19, 20]. For this purpose, a complete surgical setup was installed for a male human cadaver. The cadaver was placed prone and draped with a sterile surgical drape. A barrier drape was arranged at a height of 170 centimetres (cm) in order to separate the operating table from the anaesthesiologist’s workplace at the head of the table. During testing, the surgical staff (surgeon, assistant, scrub nurse, anaesthesiologist) wore water-resistant sterile surgical gowns, gloves, caps and a disposable surgical sterile helmet (Sterishild, Stryker, Mahwah, NJ, USA). A standard posterior midline approach to the thoracic spine was performed. Incision length was 20 cm. Several parts of the muscles and ligaments were preserved on the exposed bony structures. Surgical debridement was performed with a high-pressure hydro-surgery device. The remaining parts of the muscles and ligaments served as target tissue for surgical debridement (Fig. 1). Hydro-surgery debridement is performed with a thin stream of sterile saline that is forced under high pressure into an angled, sterile and disposable hand piece. The saline stream leaves an opening in the tip of the hand piece, and is immediately redirected into a suction collector tube. This creates a localised Venturi effect that allows the infected tissue to be simultaneously grasped, cut and removed [26]. For this study, we used a medium power setting (5 out of 10; 10: maximum power). The standard irrigation system of the hydro-surgery device was used, with 5000 millilitres (ml) of saline solution for irrigation. An already published, standardised test setup for operating room contamination was performed [19, 20]. Thus, the irrigation fluid was contaminated with *Staphylococcus aureus* (American Type Culture Collection [ATCC] 6538) grown in tryptic soy broth (Merck, Darmstadt, Germany) under aerobic conditions at 37 °C for 24 h. An adequate concentration of the contaminated irrigation fluid was selected to imitate average surgical site infection. The final concentration of colony-forming units (CFU) of *S. aureus* in the test solution was determined by colony count. Samples were spread out on Columbia blood agar (Becton Dickinson, Franklin Lakes, NJ, USA) and incubated at 37 °C for 48 h. The resulting concentration was 6.4 × 10^5^ CFU/mL staphylococci. For air sampling, 103 standard Petri dishes with mannitol salt agar (Merck) were exposed to the contaminated aerosol. The Petri dishes were placed in an operating room (6 × 8 m) at a height of 100 cm at regular intervals. The operating room was disinfected after each debridement, and a 2-h rest period was allowed before the next trial. Disinfection was performed according to hygiene guidelines for septic surgeries. Then the cadaver was redraped as explained above. After debridement, the Petri dishes were covered and collected. Standardised surveillance cultures of bodies and faces were taken from the following: surgeon, assistant, scrub nurse, anaesthesiologist and from the head of the cadaver. Everyone was instructed to remain in their usual working area, simulating the normal positions of the team members during spinal surgery. Movement was allowed only to the extent needed to simulate realistic debridement. After incubation of the Petri dishes at 37 °C for 48 h, the microorganisms were differentiated according to morphologic, physiologic and serologic criteria. A commercial test kit (Pastorex Staph Plus [Sanofi Pasteur Diagnostics, Chaska, MN, USA]) was used to identify *S. aureus*. For each positive culture, the position of the Petri dish during the test was recorded. After three trials, the wound was covered with a sterile disposable surgical tent (Medaxis, Aarau, Switzerland) and another three trials were performed with the high-pressure hydro-surgery device. The sterile disposable surgical tent has a transparent window to permit inspection of the workflow under the tent and serves as an additional protection against contamination (Fig. 2). Performance of each of the six trials continued until the irrigation fluid (5000 ml) was exhausted. Debridement time per trial was approximately 40 min. Data were processed using the Statistical Package for Social Sciences (SPSS13 Norusis/SPSS Inc., Chicago, IL, USA). Mean CFU values for the two setups were calculated.

## Results

**Fig. 3:**
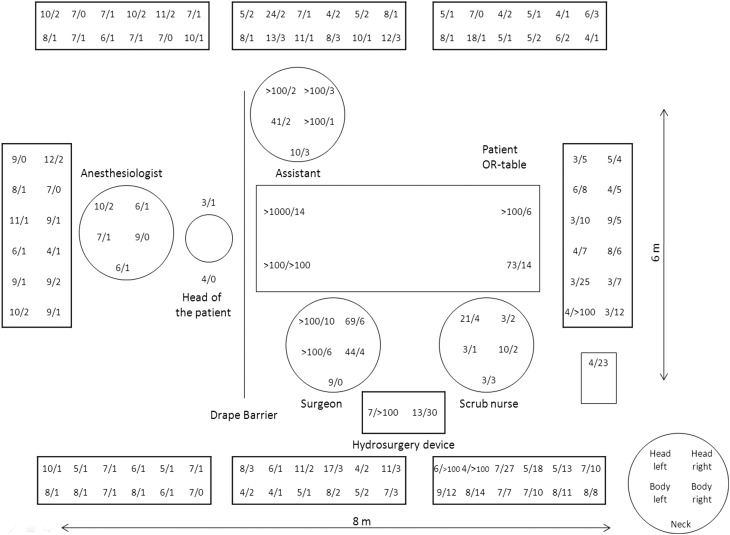
Figure showing contamination of the OR and surgical personnel (mean CFU values without (*left*) and with the surgical tent (*right*))

For both test setups, we detected environmental contamination throughout the room (6 × 8 m) (Fig. 3). For passive air sampling without the disposable surgical tent, all Petri dishes in the 6 by 8 m area showed growth of *S. aureus* (range 3 to >100 CFUs per plate). The additional surgical tent reduced the number of CFUs, and some of the Petri dishes even showed no bacterial growth. Because growth was detected in some of the most remote dishes at the room’s periphery, no correlation could be established between bacterial growth in the operating room and distance from the operating field (range 0 to >100 CFU per plate). Surveillance cultures taken without additional draping showed contamination of the faces and bodies of the surgical staff. The surgeon and the surgical assistant showed more severe contamination than did other members of the surgical team. Surveillance cultures revealed that the anaesthesiologist and the cadaver’s head to also be contaminated with *S. aureus*. For body contamination, the additional draping device produced a lower number of CFUs in the surveillance cultures for all persons present during surgery. The surgeon and the surgical assistant showed the most significant reduction in CFUs.

## Discussion

In spinal surgery, the risk of surgical site infection varies between 1 and 6% [18]. Effective treatment includes early diagnosis, parenteral antibiotics and accurate surgical debridement [18]. The hydro-surgery system enables the surgeon to precisely control the cutting, debriding and aspiration effects by adjusting the console power settings and by angulating the hand piece [26]. In this way, debridement can be performed in a more accurate and detailed manner with a decrease in surgery time. In the current study, *S. aureus* was chosen as a sample microorganism. It is easy to culture and detect as well as to differentiate from other bacteria. Contaminating the saline solution from the hydro-surgery system with *S. aureus* made it possible to evaluate the extension of the aerosol, face and body contamination with standard microbiological methods. The standardised test setup for spinal aerosol contamination used in the current study was already published in 2001 [19, 20]. Nogler et al. showed that the test setup used in the present study mirrors the intraoperative situation for infected tissue. The measured contamination was not specific to *S. aureus* and would probably be the same for any viral, bacterial or fungal agent. Surgical site infections with *S. aureus* and viral agents like hepatitis B and C and Herpes simplex following injuries with sharp and high-speed tools have been reported [7, 11, 12, 14, 22]. While the risk of airborne transmission is lower than for direct contact or injuries, there is still some risk of infection for the members of the surgical team by inhaling aerosols contaminated with pathogens such as Mycobacterium tuberculosis, legionella, hepatitis B, varicella zoster, smallpox, influenza and *S. aureus* [6, 16, 23, 27]. In addition, infections can be transmitted to patients by healthcare professionals. Such transmission was reported by Isenberg et al. [15] for a single-source outbreak of Candida tropicalis. He reported that microorganisms on the fingertips and in the nasopharynx of the scrub nurse were identified as the source of infection. Other bacterial and viral agents can survive on surfaces or instruments in the operating room and then be transmitted to medical personnel and patients [25].

In the model, a high inoculated solution was used to simulate a worst-case scenario of wound infection. The used concentration simulated the threshold of possible bacterial concentration in the wound. The current study evaluated the spread of contaminated aerosols in hydro-surgery debridement with and without an additional draping device (surgical tent). Without the surgical tent, the hydro-surgery device contaminated all individuals in the operating room (OR) and all parts of the OR to some extent. Additional protection provided by a surgical tent was seen to produce significantly less contamination of the operating theatre. The surgeon and the surgical assistant showed the greatest decrease in CFUs on their bodies. However, CFUs were found on every person in the OR in all three trials performed with the disposable surgical tent. The detected contamination was probably not related to hydro-surgery alone and most likely would be the same for other debridement procedures. Limitations of the current study are that mechanical properties and dynamics of cadaveric soft tissue might be different than those for living tissue. However, we used standardised published test setups to imitate operating room contamination in cadaveric tissue [19–21]. The findings made in the current study were similar to those obtained with surgical tools that produce potentially contaminated aerosols during spinal surgery [19, 20]. From our findings we recommend that during debridement procedures, effective body protection should be undertaken for all persons in the OR. We also recommend that the number of persons in the OR should be kept to a minimum. The personnel present in the OR should be well trained in appropriate safety procedures. To reduce the risk of contact with contaminated fluid, the surgical staff (surgeon, assistant, scrub nurse) should wear water-resistant gowns, surgical gloves and caps. Faces and eyes should be protected with surgical helmets. In addition, the anesthesiologist’s and patient’s faces should be at least covered with surgical masks and caps, or surgical helmets as well. Air filtration systems may reduce the risk of contamination for personnel and patient. We also conclude that patients with known infections should be operated at the end of the day’s surgical programme, and that the OR including all mobile equipments should be effectively disinfected after debridement procedures.

In conclusion, the current study demonstrates that contaminated aerosols produced during hydro-surgery debridement can spread all over the OR, contaminating both the animate and the inanimate environment. For hydro-surgery debridement, an additional draping device should be used to reduce the risk of inoculation with contaminated fluids. The direct infection risk for healthcare workers might be lower than the risk posed by injuries with contaminated tools. Nevertheless, a certain risk remains, especially if the contaminated aerosol is inhaled or comes into contact with conjunctival or mucous membranes.

## References

[1] Armstrong DG, Lipsky BA (2004). Diabetic foot infections: stepwise medical and surgical management. Int Wound J.

[2] Attinger CE, Bulan E, Blume PA (2000). Surgical debridement. The key to successful wound healing and reconstruction. Clin Podiatr Med Surg.

[3] Brem H, Sheehan P, Rosenberg HJ, Schneider JS, Boulton AJ (2006). Evidence-based protocol for diabetic foot ulcers. Plast Reconstr Surg.

[4] Brem H, Stojadinovic O, Diegelmann RF (2007). Molecular markers in patients with chronic wounds to guide surgical debridement. Mol Med.

[5] Crowley DJ, Kanakaris NK, Giannoudis PV (2007). Irrigation of the wounds in open fractures. J Bone Joint Surg Br.

[6] Eickhoff TC (1994). Airborne nosocomial infection: a contemporary perspective. Infect Control Hosp Epidemiol.

[7] Epstein JB, Rea G, Sibau L, Sherlock CH (1993). Rotary dental instruments and the potential risk of transmission of infection: herpes simplex virus. J Am Dent Assoc.

[8] Giachino A, Profitt A, Taine W (1988). Contamination of the conjunctiva of the orthopaedic surgeon. A technical note. J Bone Joint Surg Am.

[9] Giachino AA, Profitt AW, Taine W (1988). Expected contamination of the orthopedic surgeon’s conjunctiva. Can J Surg.

[10] Granick M, Boykin J, Gamelli R, Schultz G, Tenenhaus M (2006). Toward a common language: surgical wound bed preparation and debridement. Wound Repair Regen.

[11] Hadler SC (1990). Hepatitis B virus infection and health care workers. Vaccine.

[12] Harpaz R, Von Seidlein L, Averhoff FM (1996). Transmission of hepatitis B virus to multiple patients from a surgeon without evidence of inadequate infection control. N Engl J Med.

[13] Hassinger SM, Harding G, Wongworawat MD (2005). High-pressure pulsatile lavage propagates bacteria into soft tissue. Clin Orthop Relat Res.

[14] Hauman CH (1993). Cross-infection risks associated with high-speed dental handpieces. J Dent Assoc S Afr.

[15] Isenberg HD, Tucci V, Cintron F, Singer C, Weinstein GS, Tyras DH (1989). Single-source outbreak of Candida tropicalis complicating coronary bypass surgery. J Clin Microbiol.

[16] Legnani P, Checchi L, Pelliccioni GA, D’Achille C (1994). Atmospheric contamination during dental procedures. Quintessence Int.

[17] Lipsky BA, Berendt AR, Deery HG (2004). Diagnosis and treatment of diabetic foot infections. Clin Infect Dis.

[18] Massie JB, Heller JG, Abitbol JJ, McPherson D, Garfin SR (1992). Postoperative posterior spinal wound infections. Clin Orthop Relat Res.

[19] Nogler M, Lass-Florl C, Ogon M, Mayr E, Bach C, Wimmer C (2001). Environmental and body contamination through aerosols produced by high-speed cutters in lumbar spine surgery. Spine (Phila Pa 1976).

[20] Nogler M, Lass-Florl C, Wimmer C, Bach C, Kaufmann C, Ogon M (2001). Aerosols produced by high-speed cutters in cervical spine surgery: extent of environmental contamination. Eur Spine J.

[21] Nogler M, Lass-Florl C, Wimmer C, Mayr E, Bach C, Ogon M (2003). Contamination during removal of cement in revision hip arthroplasty. A cadaver study using ultrasound and high-speed cutters. J Bone Joint Surg Br.

[22] Quebbeman EJ, Telford GL, Hubbard S (1991). Risk of blood contamination and injury to operating room personnel. Ann Surg.

[23] Reboli AC, John JF, Platt CG, Cantey JR (1990). Methicillin-resistant *Staphylococcus aureus* outbreak at a Veterans’ Affairs Medical Center: importance of carriage of the organism by hospital personnel. Infect Control Hosp Epidemiol.

[24] Tabor OB, Bosse MJ, Hudson MC (1998). Does bacteremia occur during high pressure lavage of contaminated wounds?. Clin Orthop Relat Res.

[25] Talon D (1999). The role of the hospital environment in the epidemiology of multi-resistant bacteria. J Hosp Infect.

[26] Vanwijck R, Kaba L, Boland S, Gonzales y Azero M, Delange A, Tourbach S (2010). Immediate skin grafting of sub-acute and chronic wounds debrided by hydrosurgery. J Plast Reconstr Aesthet Surg.

[27] Walter CW, Kundsin RB, Brubaker MM (1963). The incidence of airborne wound infection during operation. JAMA.

[28] Watanabe M, Sakai D, Matsuyama D, Yamamoto Y, Sato M, Mochida J (2010). Risk factors for surgical site infection following spine surgery: efficacy of intraoperative saline irrigation. J Neurosurg Spine.

